# A Study on the Materials Used in Ancient Wooden Architectural Paintings at DaZhong Gate in Confucius Temple, Qufu, Shandong, China

**DOI:** 10.3390/ma17092170

**Published:** 2024-05-06

**Authors:** Kuiju Li, Kezhu Han, Gele Teri, Yuxiao Tian, Menglei Cui, Yunpeng Qi, Yuhu Li

**Affiliations:** 1 Engineering Research Center of Historical Cultural Heritage Conservation, Ministry of Education, School of Materials Science and Engineering, Shaanxi Normal University, Xi’an 710119, China; kuijuli@163.com (K.L.); hankekezhu@126.com (K.H.); tianyuxiaotyx@163.com (Y.T.); 2 Northwest Nonferrous Survey and Engineering Company, Xi’an 710000, China

**Keywords:** ancient wooden architecture, color painting, pigments, binders, technical analysis

## Abstract

This study analyzes the pigments and binders used in the painted wooden structure of DaZhong Gate in the Confucius Temple in Qufu, Shandong Province, China. Five samples were collected from the building and analyzed using techniques such as polarized light microscopy (PLM), energy-dispersive X-ray spectroscopy (EDX), micro-Raman spectroscopy (m-RS), and Fourier-transform infrared spectroscopy (FT-IR). The findings reveal that the red, yellow, green, and blue pigments are identified as lead red, lead chromate yellow, emerald green, and ultramarine, respectively. The white pigment is determined to be a combination of chalk and lead white or anglesite. Considering the production period of the yellow and green pigments, it is inferred that architectural paintings underwent restoration or repainting during the late Qing Dynasty. The analysis of the binder in the pigment using pyrolysis–gas chromatography/mass spectrometry (Py-GC/MS) reveals that the binder employed is a protein-based glue. Additionally, the detected presence of Heat-bodied tung oil suggests a potential connection to traditional Chinese painting techniques on wooden surfaces. This discovery not only contributes to the historical research of the Confucius Temple but also provides crucial data for the conservation and restoration efforts of this culturally significant heritage site.

## 1. Introduction

**Figure 1:**
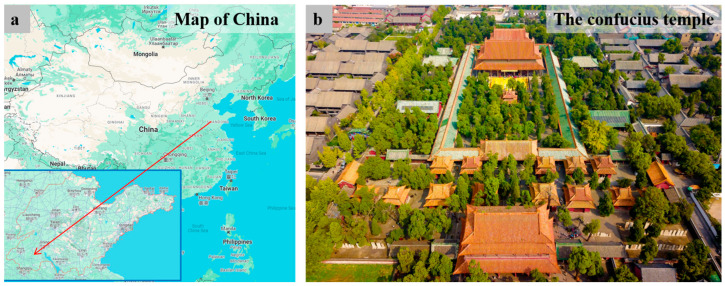
(**a**) The location of the Confucius Temple in Qufu City, Shandong Province; (**b**) an aerial view of the Confucius Temple (figure).

The Confucius Temple, situated in Qufu City, Shandong Province, China (Figure 1), was originally erected in the year 479 BCE. This shrine venerates Confucius, the esteemed ancient Chinese philosopher and educator. The temple’s design mirrors Confucius’s dwelling and adheres to the architectural standards of an imperial palace. It stands as one of the four major ancient architectural complexes in China, holding considerable significance in world architectural history. The temple is of profound historical and cultural importance, possessing artistic value as well. In 1191 CE, the Confucius Temple experienced substantial expansion through the construction of additional structures, such as the DaZhong Gate. In the second year of the Yongzheng reign of the Qing Dynasty (1724 AD), the Temple of Confucius in Qufu was destroyed by lightning fire, and it took six years to rebuild it. In 1959, the partial restoration of the painted decorations on buildings such as the Shengshi Gate, DaZhong Gate, and Shengji Hall of the Temple of Confucius in Qufu was carried out. Over the centuries, the temple has undergone numerous expansions and renovations, ensuring its preservation to the present day.

The DaZhong Gate, situated within the Confucius Temple’s ancient architectural complex in Qufu, is adorned with a plethora of architectural paintings. Ancient Chinese architectural paintings encompass the artistic practice of embellishing buildings with colored paintings, reflecting a rich tradition with roots extending back to the Spring and Autumn Period. Historical records from this era mention the application of red paint to wooden structures. This artistic form evolved and matured across various dynasties, including the Qin, Han, Wei, Jin, Southern and Northern dynasties, Sui, Tang, Song, Yuan, and beyond [1]. The pinnacle of architectural paintings was reached during the Ming and Qing dynasties [1]. Commonly adorning structures such as temples, palaces, and government buildings, these paintings typically depict various themes like floral patterns, dragons and phoenixes, figures, stories, landscapes, and more. Beyond their decorative function, these artworks serve reflective purposes, offering glimpses into the cultural characteristics of the society during the respective periods.

The ancient Chinese architectural paintings extensively utilized natural and probably artificial mineral pigments [2,3]. For instance, the more widely used red pigments are cinnabar (HgS) [4], hematite (α-Fe_2_O_3_), red lead (Pb_3_O_4_), and synthetic red lead; the yellow pigments are orpiment (As_2_S_3_), realgar (As_4_S_4_) [1], and goethite (FeO·OH); the green colors are malachite [CuCO_3_·Cu(OH)_2_] [5], emerald green [Cu(CH_3_COO)_2_·3Cu(AsO_2_)_2_], and atacamite [Cu_2_(OH)_3_Cl]; the blue pigments are lapis lazuli [(Na,Ca)_8_(AlSiO_4_)_6_(S,Cl)_2_], Chinese blue (BaCuSi_4_O_10_), and azurite [2CuCO_3_·Cu(OH)_2_]; the white pigments are lead white [2PbCO_3_·Pb(OH)_2_], synthetic lead white, chalk (CaCO_3_), and dolomite [CaMg(CO_3_)_4_]; and the black pigments include carbon black (C) and magnetite (Fe_3_O_4_). In the Du Le Temple of the Liao Dynasty (10th century AD), materials such as carbon black, cinnabar, lapis lazuli, massicot (β-PbO), litharge (α-PbO), and atacamite were used [6]. In the Longju Temple of the Ming Dynasty (15th century AD), materials like atacamite, azurite, carbon black, and cinnabar were utilized [4]. In the Taiping Heavenly Kingdom Prince Dai’s Mansion during the Qing Dynasty (19th century AD), materials such as cinnabar, ivory black (C), indigo blue (C_16_H_8_N_2_Na_2_O_8_S_2_) and phthalocyanine green (CuC_32_N_8_Cl_16_) were employed [3]. To express it clearly, some commonly used pigments are listed in Table 1. To ensure adherence for an extended period on the plaster of wooden structures or silica-based walls, these mineral pigments were mixed with binders. Binders typically consisted of natural organic substances like animal skin glue, egg white, casein, peach gum, and linseed oil [7]. For instance, the binder of the mineral pigments used in Qin Shihuang’s Terracotta Warriors (3rd century BC) included animal glue and egg whites [8,9]. Animal glue was also found in the marinade layer of the Western Han Dynasty (3rd century BC) painted pottery terracotta warriors [10]. Plaster, a traditional Chinese civil engineering technique, was applied to the surface of wooden structures to prevent decay and moisture while providing a substrate for painting [11]. The plaster for painting ancient wooden buildings typically comprised tung oil, flour, lime water, brick powder, and plant fiber. In Qing Dynasty’s Buddhist Temple in Beijing (17th century AD), the plaster layer incorporated brick powder and ramie fibers [12]. The Yi Ma Wu Hui layer of Putuo Zongcheng Temple (18th century AD) utilized tung oil, gray brick powder, and ramie fiber [11].

The analysis of mineral pigments entails a thorough examination across three dimensions: morphology observation, elemental determination, and composition identification [3]. The utilization of polarized light microscopy (PLM) [3,13] facilitates the observation and assessment of morphological intricacies, optical properties, and crystalline features of mineral pigments. Advanced techniques, including energy-dispersive X-ray spectroscopy (EDX) [14], scanning electron microscopy with energy-dispersive spectroscopy (SEM-EDS) [15], and energy-dispersive X-ray fluorescence (XRF) [16], are employed to determine the elemental constitution of the pigments. Moreover, sophisticated methods like X-ray diffraction (XRD) [17], Fourier-transform infrared spectroscopy (FT-IR) [6], and micro-Raman spectroscopy (m-RS) [18] further delineate the compositional constituents of these pigments. Throughout this investigation, we employed a combination of analytical techniques for the identification of pigment components. PLM, EDX, m-RS, and FT-IR were utilized to discern the constituents of the pigments. Simultaneously, pyrolysis–gas chromatography/mass spectrometry (Py-GC/MS) [19] was applied to ascertain the composition of binders within the pigments. This comprehensive approach ensures a thorough understanding of both the physical and chemical characteristics of the mineral pigments and their binder components.

In the current study, small samples of pigments were collected from the ancient architectural color paintings of the DaZhong Gate in the Confucius Temple, Qufu. Our investigation involved a thorough analysis of the components present in both the pigments and binders used in these historical artworks.

## 2. Materials and Methods

### 2.1. Pigment Sample Information

**Figure 2:**
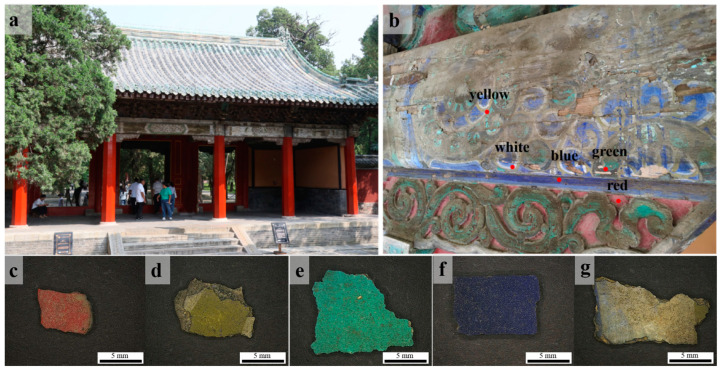
(**a**) The DaZhong Gate of the Confucius Temple; (**b**) marked positions of the sampling points inside the building; photographs of the sampled pigments: (**c**) DZ-1 (red), (**d**) DZ-2 (yellow), (**e**) DZ-3 (green), (**f**) DZ-4 (blue), and (**g**) DZ-5 (white). The length of the scale bar in the image is 5 mm.

On the inner side of the wooden main beam in the DaZhong Gate of the Confucius Temple, as shown in Figure 2, we collected samples displaying five distinct colors: red, yellow, green, blue, and white. There is a scale bar with a length of 5 mm in the figure, and the area of the sample is approximately 1 cm^2^. All five samples had dirt that was difficult to remove. To enhance the conservation of the color paintings, each sampled segment was meticulously chosen from regions either already detached or showing signs of imminent detachment.

### 2.2. Experimental Methods and Instrumentation

#### 2.2.1. Polarized Light Microscopy (PLM)

The samples were initially subjected to pretreatment, where pigment particles were delicately scraped from the specimen using a surgical blade. These particles were then added to alcohol and dispersed for 30 min using an ultrasonic disperser. The dispersed pigment suspension was dropped onto a glass slide, and after the alcohol evaporated, a cover slip was placed over it. Subsequently, the entire slide was heated to 60–70 °C on a temperature-controlled heater, allowing the resin (Meltmount, Cargille; Cedar Grove, NJ, USA; refractive index of 1.662) to melt and permeate the entire cover slip. The cover slip was then slid along one side to secure the pigment particles.

Observations were carried out employing a polarized light microscope (Olympus BX53M, Shinjuku, Japan) with a magnification of 50 × 10.

#### 2.2.2. Energy-Dispersive X-ray Spectroscopy (EDX)

The samples were directly placed on the sample stage for testing in ambient conditions, with each sample requiring approximately 5 min for analysis. The corresponding results are shown in Table 2.

Energy-dispersive X-ray spectroscopy (EDX, EDX-7000, Shimadzu, Kyoto, Japan) was employed for the elemental characterization of the pigments. The X-ray tube within the EDX system comprised a rhodium target and a silicon drift detector, enabling the detection of various elements spanning from sodium (Na) to uranium (U).

#### 2.2.3. Micro-Raman Spectroscopy (m-RS)

The samples were directly placed on glass slides and positioned on the sample stage. Utilizing a microscope system, crystal particles within the samples were located, and Raman spectra were acquired using the Renishaw Invia reflection system (Invia Reflex, Renishaw, UK).

Micro-Raman spectroscopy was employed for the spectral analysis of pigment samples. It utilized a grating with 400 lines/mm and a 2-micron spot size, covering a spectral range from 100 cm^−1^ to 3500 cm^−1^. Excitation wavelengths of 532 nm and 785 nm were available. The objective had a magnification of 50×, with an exposure time of 30 s, an accumulation time of 1 s, and a laser power of 2 mW. The Renishaw Wire software version 4.3 (Wotton-under-Edge, UK) was used for spectrum processing equipped with an argon ion laser, Leica microscopes, and a charge-coupled detector (CCD).

#### 2.2.4. Fourier-Transform Infrared Spectroscopy (FT-IR)

Potassium bromide (KBr) was placed in a drying oven at 300 °C for 3 h. Pigment particles were delicately scraped from the specimen using a surgical blade. Subsequently, the scraped pigment particles weighing 2 mg in total were mixed and ground with 150 mg of dried potassium bromide, and the resulting mixture was pressed into a thin plate using a hydraulic press.

Fourier-transform infrared spectroscopy (FT-IR, Thermo Scientific Nicolet iS10, Waltham, MA, USA) was employed to scan and detect the pretreated sample pellets in the wavelength range of 500–4000 cm^−1^. The spectral resolution was set at 1 cm^−1^.

#### 2.2.5. Pyrolysis–Gas Chromatography/Mass Spectrometry (Py-GC/MS)

An amount of 0.1 mg of the sample was taken, powdered, and placed into a thermally cleaved sample cup. Then, 2 μL of a 25% mass fraction solution of tetramethylammonium hydroxide (TMAH, Aladdin, Shanghai, China) was added and allowed to precipitate for 1 h. Subsequently, the mixture was placed under an infrared lamp and left to cleave after water evaporation. During the analysis, the pyrolysis temperature was set at 600 °C, the pyrolysis interface temperature was 300 °C, and the pyrolysis inlet temperature was 250 °C. The 40 °C chromatographic column was ramped to 280 °C at a rate of 10 °C/min, and this rate was maintained for 20 min. In the GC/MS procedure, a high-purity helium carrier gas was used with an inlet pressure of 15.2 kPa and a split ratio of 1:100. A constant flow mode was maintained in the electron pressure control system. The mass spectrometer was run with electron ionization at an ionization energy of 70 eV. The scanning range was set from 50 to 750, with a cycle time of 0.5 s. Subsequently, we used NIST14 and the corresponding mass spectrometer to identify the separated substances.

The pyrolysis–gas chromatography/mass spectrometry (Py-GC/MS) technique involved the use of a pyrolysis unit (EGA/PY-3030D, Frontier Labs, Fukushima, Japan) and a gas chromatography/mass spectrometry instrument (GC/MS-QP2010Ultra, Shimadzu, Japan). The chromatographic column utilized in this method was SLB-SMS (5% diphenyl/95% dimethyl siloxane), which has a length of 30 m, an inner diameter of 0.25 mm, and a film thickness of 0.23 mm.

## 3. Results and Discussion

### 3.1. Pigments

#### 3.1.1. Red

**Figure 3:**
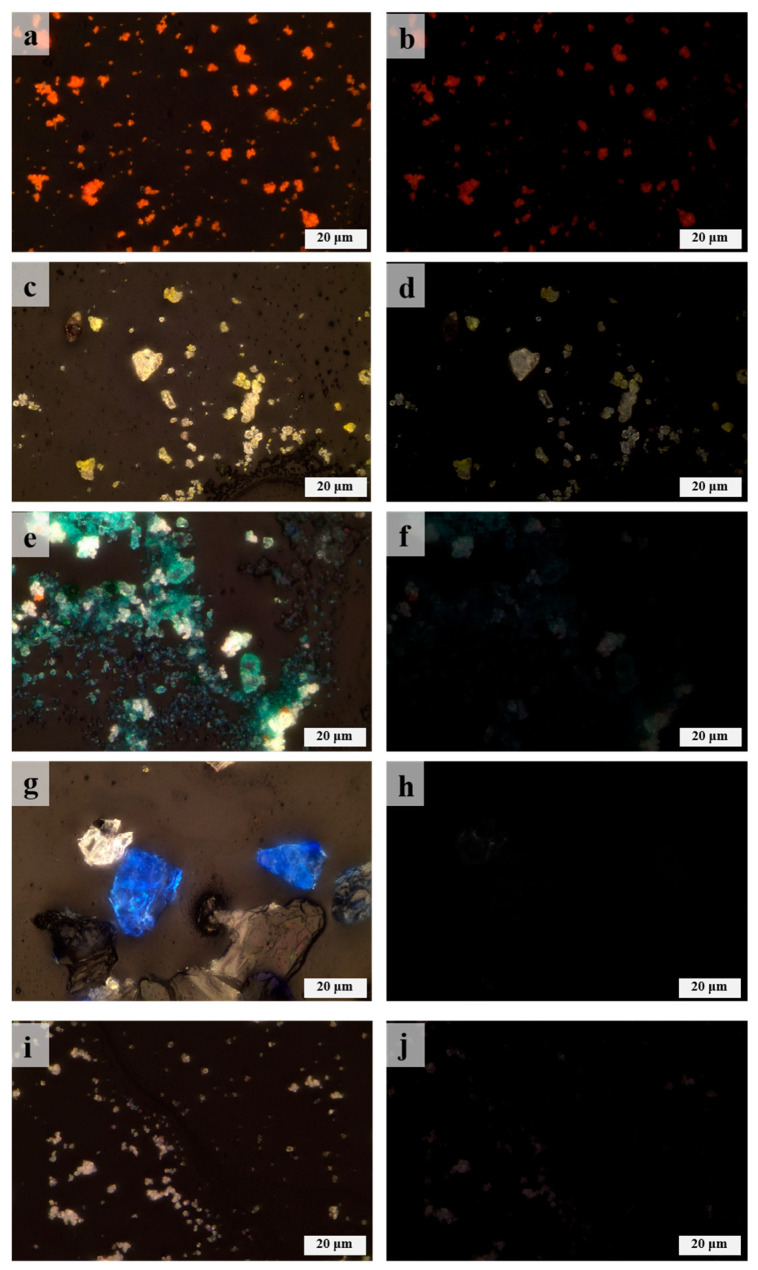
PLM images observed under single and orthogonally polarized light for (**a**,**b**) red, (**c**,**d**) yellow, (**e**,**f**) green, (**g**,**h**) blue, and (**i**,**j**) white pigments. All samples were observed under 100 × 5 magnifications. The sampling positions are shown in Figure 2.

**Figure 4:**
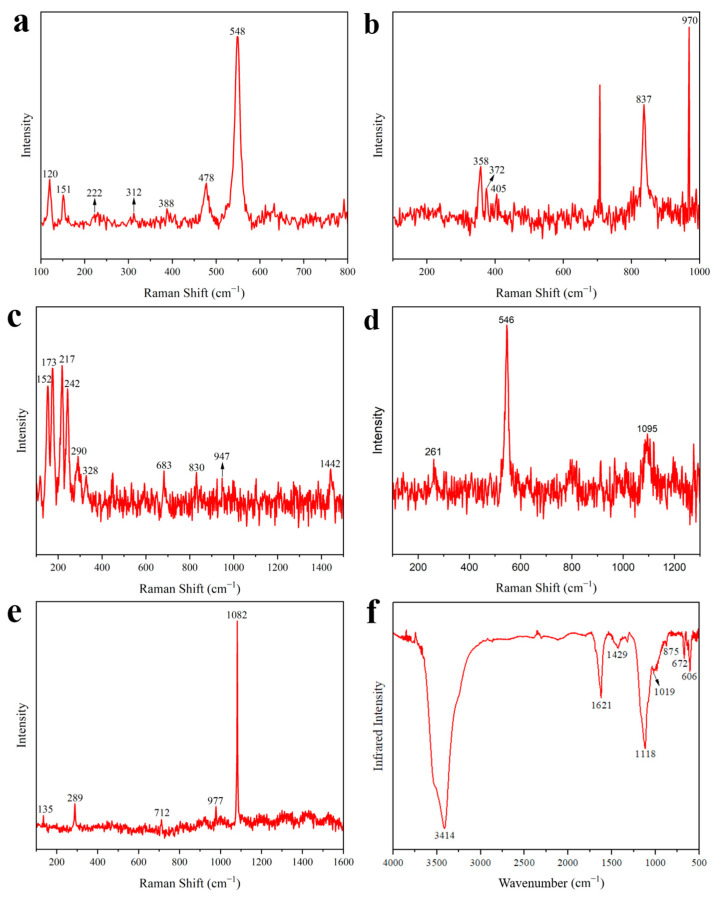
(**a**) Raman spectrum for red pigment (DZ-1); (**b**) Raman spectrum for yellow pigment (DZ-2); (**c**) Raman spectrum for green pigment (DZ-3); (**d**) Raman spectrum for blue pigment (DZ-4); (**e**) Raman spectrum of white pigment (DZ-5); (**f**) FT-IR spectrum for white pigment (DZ-5).

The morphology of the DZ-1 sample particles was observed using PLM. As illustrated in Figure 3a,b, under single polarized light (plane-polarized light), the pigment particles exhibited an indistinct crystalline edge. Under orthogonally polarized light (cross-polarized light), the pigment particles displayed strong extinction. Considering these characteristics, we preliminarily deduce that its optical properties belong to either cinnabar or red lead (Pb_3_O_4_) [20]. Further, EDX elemental analysis results (Table 2) revealed a significant amount of lead in the DZ-1 sample, confirming its definite content of red lead. None of the other red pigments contain lead elements, and the sample lacks characteristic elements of cinnabar as well. Analyzing the sample with m-RS, as shown in Figure 4a, peaks were observed in the Raman spectrum at 120, 151, 222, 312, 388, 478, and 548 cm^−1^. Comparing these peaks with standard Raman spectra of red pigments, we can see that they align precisely with the Raman spectrum of red lead [21,22,23,24,25]. Notably, the peak at 548 cm^−1^ is attributed to the Pb–O stretching vibration in red lead [26].

Red lead, known as lead tetroxide or minium, is one of the most commonly used red pigments in ancient China. The manufacturing process of red lead has been documented in China since the 3rd century BCE, and records about red lead can also be found in Roman literature from the 1st century AD. The DZ-1 sample exhibits localized darkening on its surface under a macro lens, as shown in Figure 2c, a characteristic feature of red lead distinct from other red pigments. Due to its lively chemical nature, ancient lead-containing pigments, such as red lead, are prone to environmental erosion and can easily discolor into plattnerite (PbO_2_) during prolonged preservation. Reports of red lead aging into lead dioxide have been documented in the Dunhuang Mogao Caves in China [27] and Panselinos’ Byzantine wall paintings in Greece [28]. Red lead may also contain other substances similar to massicot. The ancient Chinese production of red lead involved heating lead, and as the temperature increased, metallic lead would first react with oxygen in the air to form massicot (β-PbO). When the temperature reached 450–470 °C, it would further oxidize into the red pigment red lead [29,30]. Consequently, some red lead pigments may contain a mixture of massicot.

#### 3.1.2. Yellow

The elements detected in the sample using EDX include Pb and Cr (Table 2), and based on the elemental composition, it corresponds to the yellow pigment lead chromate yellow [22]. However, the EDX analysis results show the presence of Ca, Ba, and Zn. Based on their chemical formulas, they may correspond to barium chromate and zinc chromate. Barium chromate typically exhibits strong peaks at 863 cm^−1^ and medium-intensity peaks at 901 cm^−1^ in Raman spectra [22]. Zinc chromate usually shows a strong peak at 872 cm^−1^ and medium-intensity peaks at 343 cm^−1^ and 941 cm^−1^ in Raman spectra [22]. As shown in Figure 4b, none of these peaks were observed in the Raman detection results of sample DZ-2. Therefore, we speculate that the existence of these two pigments is unlikely. Additionally, barium chromate and zinc chromate are not commonly found in the pigments used in ancient Chinese architectural paintings. Moreover, there are some stains on the surface of sample DZ-2 that are difficult to remove, so it is inferred that Ca, Ba, and Zn are impurities in the sample. Figure 3c,d show particle photos of the DZ-2 sample under single polarized light and orthogonally polarized light. As shown in Figure 4b, in the m-RS analysis of DZ-2, peaks appear at 358, 372, 405, and 837 cm^−1^. Comparing with the standard Raman spectroscopy database, these peaks correspond to chrome yellow deep (PbCrO_4_·PbO) [1,22,24]. The strong peak at 970 cm^−1^ possibly corresponds to lead white [2PbCO_3_·Pb(OH)_2_] or anglesite (PbSO_4_), and in the DZ-2 sample, it is located near the junction of the yellow and white areas, suggesting a possible mixture of white pigment. It is also possible that the yellow and white pigments were mixed to achieve the desired color by the painter.

Chrome yellow deep is the aged form of lead chromate yellow, also known as chrome yellow. Lead chromate yellow was first synthesized by the French chemist L.N. Vauquelin in 1809, and its industrial production began in Germany in 1818. It has been discovered in Ming Dynasty polychrome sculptures at the Chongyang Temple in Shanxi Province, China [31]. As this pigment was discovered relatively late, the author suggests that the painted decorations on the DaZhong Gate of the Confucius Temple might have undergone restoration and repainting during the late Qing Dynasty.

#### 3.1.3. Green

Using PLM to observe the DZ-3 sample, as shown in Figure 3e,f, it can be seen that under single polarized light, it presents a fan-shaped or even circular-fan surface shape, some even showing a rounded and angular shape. Under orthogonally polarized light, it exhibits strong extinction. Its color is a vibrant blue-green. Through EDX testing (Table 2), it is observed that the content of As and Cu elements is particularly high. Combined with the characteristics observed under PLM and the results of EDX analysis, it is preliminarily determined that DZ-3 contains emerald green [Cu(CH_3_COO)_2_·3Cu(AsO_2_)_2_] [13,20]. Analyzing DZ-3 with m-RS, the obtained spectrum in Figure 4c shows strong peaks at 152, 173, 217, and 242 cm^−1^, and relatively weaker peaks at 290, 328, 683, 830, 947, and 1442 cm^−1^. Compared with the standard Raman spectrum of emerald green, it is found that these peaks correspond to the standard spectrum [22]. Therefore, we can confirm that the green pigment in DZ-3 is emerald green. Emerald green exhibits numerous peaks in the 100–400 cm^−1^ wavelength range, originating from the vibrations of Cu–O and As–O [32]. The peaks at 947 and 1442 cm^−1^ correspond to the acetate groups C–C and –CO_2_ in emerald green [33].

Emerald green is a synthetically produced pigment that is highly toxic, and it tends to turn black upon exposure to hydrogen sulfide (HS). It was first synthesized in Germany in 1814 [34] and was introduced to China in the mid-19th century [35]. It was widely used in the ancient architectural paintings of late Qing Dynasty China. Examples of its use can be found in the Wuying Hall of the Imperial Palace in Beijing, China [35], and the Summer Palace in Beijing [36], China. According to the appearance time of this green pigment, the authors believe that the use of emerald green suggests that the architectural paintings might have undergone restoration and repainting during the late Qing Dynasty.

#### 3.1.4. Blue

The polarization characteristics of DZ-4 were observed using PLM. As shown in Figure 3g, under single polarized light, the pigment particles exhibited mineral angular shapes and a vivid deep blue color. As shown in Figure 3h, under orthogonally polarized light, there was a strong extinction effect, even complete extinction. Leveraging the characteristic of full extinction under PLM, among common blue pigments, it can be tentatively inferred that the pigment used is ultramarine [20]. Further analysis using EDX on sample DZ-4 revealed a significant amount of Ca, S, and Si (Table 2). Comparing the chemical formulas of blue pigments, only ultramarine and Egyptian blue (CaCuSi_4_O_10_) roughly matched these elements; in fact, Egyptian blue was not found, since no copper was detected. Performing m-RS analysis on the sample, the Raman spectrum in Figure 4d shows a strong peak at 546 cm^−1^, with weaker peaks at 261 and 1095 cm^−1^. Compared with the standard Raman spectrum, it is consistent with the characteristic peaks of ultramarine [22]. It is worth noting that the sulfur-free radical, mainly S^3−^, as the chromophore in ultramarine produces a strong Raman band at 544 cm^−1^ [35]. Combining PLM, EDX, and m-RS, it can be confirmed that the blue pigment used in DZ-4 is ultramarine.

Ultramarine blue comprises both natural ultramarine (lapis lazuli) [(Na,Ca)_8_(AlSiO_4_)_6_(S,Cl)_2_] and synthetic ultramarine (Na_7_Al_6_Si_6_O_24_S_3_), making them challenging to distinguish through Raman spectroscopy [37]. The first synthetic ultramarine was produced in 1828. Due to its complex chemical structure, a fully quantitative analysis method was not established until the early 19th century to elucidate the chemical composition of ultramarine. Ultramarine tends to fade and decompose under acidic or alkaline conditions, even at low acid concentrations over an extended period. It has been extensively used in ancient Chinese architectural color paintings, such as found in Tanxi Hall of the Forbidden City in Beijing, China [38], and Tongcai paintings in Guangzhou, China [39].

#### 3.1.5. White

EDX spectrum analysis was performed on the white sample DZ-5, revealing a significant presence of Ca, S, and Ba elements (Table 2). Comparing the elemental composition of white pigments, chalk (CaCO_3_) and gypsum (CaSO_4_) primarily contain Ca, while barite (BaSO_4_) contains Ba. The sample may also contain white pigments with Zn and Pb. As shown in Figure 3i,j, the PLM analysis of DZ-5 displays pronounced extinction under orthogonally polarized light. The m-RS analysis of DZ-5 yielded a Raman spectrum, shown in Figure 4e, featuring a super-strong peak at 1082 cm^−1^ and relatively weaker peaks at 132, 289, 712, and 977 cm^−1^. Compared with the standard Raman spectra of white pigments, these peaks correspond to those of chalk [22]. Specifically, 1082 cm^−1^ corresponds to the ν_1_ symmetric stretching vibration of CO_3_^2−^; 289 cm^−1^ corresponds to the motion of Ca^2+^ relative to the CO_3_^2−^ group; and 712 cm^−1^ is attributed to the ν_4_ symmetric deformation vibration of CO_3_^2−^ [40,41]. Peaks at 135 and 977 cm^−1^ correspond to the standard spectrum of lead white [2PbCO_3_·Pb(OH)_2_] or anglesite (PbSO_4_) [22,42]. Additionally, as shown in Figure 4b, the Raman spectrum of DZ-2 shows a peak at 970 cm^−1^, consistent with the peak of lead white or anglesite [22]. Since the yellow and white regions in DZ-2 are adjacent and considering the presence of Pb and S in the EDX analysis of the white sample, it is plausible that the white sample contains lead white or anglesite. The FT-IR analysis of the white sample DZ-5, as shown in Figure 4f, indicates the presence of OH^−^, CO_3_^2−^, and SO_4_^2−^ groups. Specifically, 1429 is attributed to the ν_3_ and anti-symmetric stretching vibrations of carbonate ions [26,43,44]; 875 cm^−1^ corresponds to the out-of-plane deformation vibration of CO_3_^2−^ [45]; 1118 cm^−1^ corresponds to the S–O anti-symmetric stretching vibration in SO_4_^2−^ [46]; 606 cm^−1^ corresponds to the S–O anti-symmetric bending vibration [46]; 3414 cm^−1^ corresponds to the OH stretching vibration [26]; and 1019 cm^−1^ corresponds to the OH bending vibration [26]. Considering all these analyses, it can be inferred that the white pigment in DZ-5 is predominantly chalk, with a small amount of lead white or anglesite mixed pigment and possibly other SO_4_^2−^-containing white pigments. Unfortunately, the complete Raman spectra of lead white or anglesite, as well as other SO_4_^2−^-containing white pigments, were not obtained during the white sample analysis.

Chalk and lead white were commonly used painting pigments in ancient China. The combination of various pigments in ancient Chinese architectural paintings was also common. Lead white is a lead-containing pigment that easily transforms into black lead dioxide (PbO_2_) in the atmospheric environment, causing a darkening of color. The absence of black color in DZ-5 suggests that the low content of lead white in the mixed pigments may contribute to this phenomenon.

### 3.2. Binder

**Figure 5:**
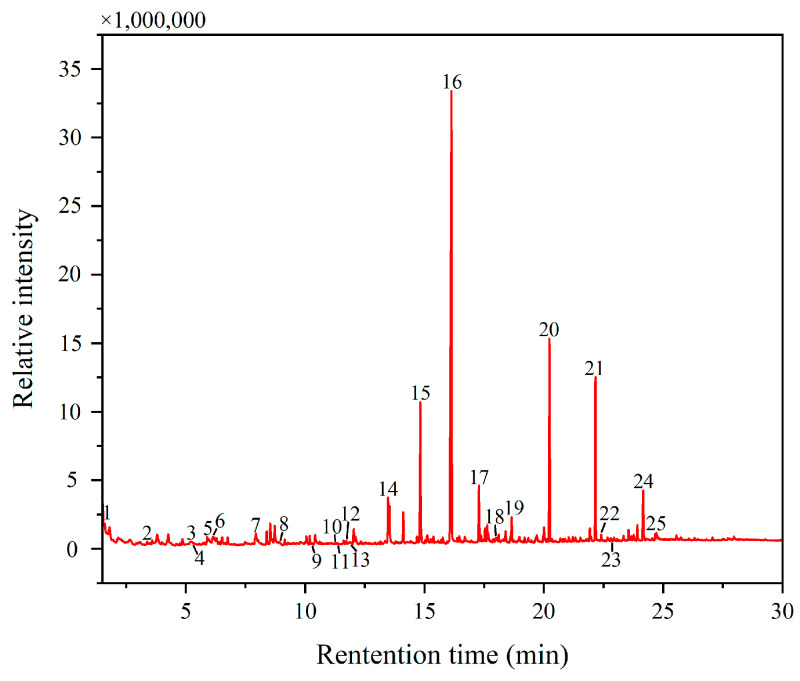
The total ion chromatogram (TIC) of the yellow pigment samples (DZ-2).

Using Py-GC/MS, an analysis was conducted on the yellow pigment sample DZ-2. The total ion chromatogram (TIC) of the analysis results is depicted in Figure 5, with representative and major peaks marked and listed in Table 3. The presence of amino acids such as glycine (peak 3), alanine (peak 6), valine (peak 7), L-proline, 1-methyl-, methyl ester (peak 8), hydroxyproline (peak 13), and some nitrogen-containing substances like methylamine, N,N-dimethyl- (peak 1), 1H-pyrrole, 1-methyl- (peak 2), methyl 1-methylpyrrole-2-carboxylate (peak 9), and benzene, isocyanato (peak 23) indicates the presence of proteins in DZ-2 [19], suggesting the existence of protein-based binders. The presence of naphthalene (peak 10) and 1H-indene, 1-methylene- (peak 11) also supports the presence of proteins [26]. The detected hydroxyproline in the sample is a characteristic amino acid of animal collagen [36]. Additionally, protein (peak 12) and protein blood and glue (peak 18) were directly detected in the sample. Furthermore, as shown in Figure 4f, in the infrared detection results of the white pigment sample, the absorption peak at 1621 cm^−1^ can be attributed to the amide Ⅰ band, which is an important component of amino acids in proteins. Considering all the analyses, the binder used in the pigments primarily contains proteins. Due to prolonged exposure to outdoor conditions, the binder has undergone significant aging, resulting in a decrease in protein content compared to a fresh binder. The application of methylation technology is the reason why the test results contain a large number of methyl ester compounds, such as octanedioic acid, dimethyl ester (peak 15), nonanedioic acid, and dimethyl ester (peak 16). Since pigment binders on the same artwork are generally consistent [3,47], we focused our analysis on the pigment binder of the yellow sample in this study.

**Figure 6:**
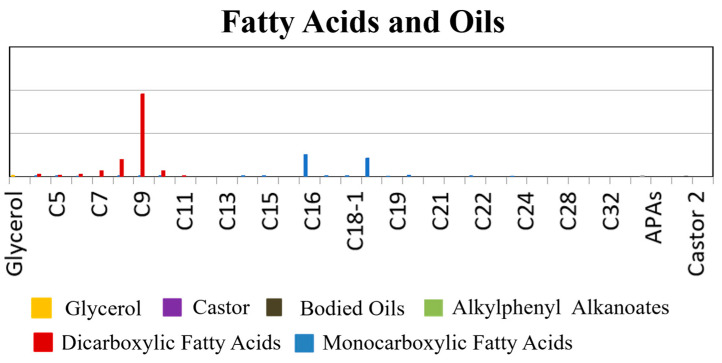
The relative concentrations of fatty acids for the yellow pigment sample (DZ-2) obtained by Py-GC/MS; carboxylic acid is shown with carbon numbers of n. The very small amounts of substances detected in the sample are not apparent in the figure.

Simultaneously, Py-GC/MS was employed to detect the fatty acids in the DZ-2 sample, and the results are presented in Figure 6. Both monobasic carboxylic fatty acids and dibasic carboxylic fatty acids were identified in the sample. Additionally, substances classified as alkylphenyl alkanoates (APAs) were detected, suggesting the presence of heat-bodied tung oil [19,26,48]. The ratio of palmitic acid to stearic acid (P/S) is usually used to distinguish various types of dry oil [49]. The P/S value detected in the sample was 1.19, further validating the speculation. Heat-bodied tung oil was obtained by heating or refining tung oil before use. Furthermore, the test results revealed a small amount of aged glue containing proteins, confirming the presence of animal glue.

## 4. Conclusions

This study involved a comprehensive analysis of five pigment samples collected from the peeling areas of the DaZhong Gate in the Confucius Temple in Qufu. The techniques used included PLM, EDX, m-RS, and FT-IR. The results revealed that the red pigment is lead red, the yellow pigment is lead chromate yellow, the green pigment is emerald green, the blue pigment is ultramarine, and the white pigment is a mixture of chalk and lead white or anglesite. Lead red, ultramarine, chalk, and lead white are commonly used pigments in ancient Chinese architecture. It is noteworthy that lead chromate yellow and emerald green, which appeared in 1809 and 1814, respectively, were extensively used in architectural paintings during the late Qing Dynasty. Therefore, it is speculated that the architectural paintings of DaZhong Gate underwent restoration or repainting during the late Qing Dynasty.

The analysis of the Py-GC/MS detection results indicates that the binder used in the pigments contains protein-based glue, and based on the characteristic amino acid hydroxyproline, it is speculated that it may contain animal glue. The presence of fatty acids in the detection results suggests the inclusion of heat-bodied tung oil in the pigments. The occurrence of drying oil can be explained by the traditional Chinese painting technique on wooden surfaces. Before applying the pigment layer, a layer of heat-bodied tung oil was brushed on the plaster, and over time, it mixed with pigments and glue during the painting process.

The study of the pigments and binders used in the architectural paintings of DaZhong Gate in the Confucius Temple provides a universal method for the future identification of components in ancient architectural paintings. This research contributes data and evidence to support the conservation and better management of the architectural paintings in the DaZhong Gate of the Confucius Temple. Additionally, more research on the preservation status of other paintings is needed for more effective preventive and intervention-based conservation efforts.

## Figures and Tables

**Table 1:** The examples of usage and the enumeration of common pigments mentioned in the Introduction.

Color	Pigment Name	Chemical Formula	Location of Use
Red	Cinnabar	HgS	Du Le Temple (10th century AD), etc.
	Red lead	Pb_3_O_4_	Royal Residence (19th century AD)
	Litharge	α-PbO	Du Le Temple (10th century AD)
Yellow	Orpiment	As_2_S_3_	Assembly Hall (19th century)
	Realgar	As_4_S_4_	Royal Taoist Temple (16th century)
	Massicot	β-PbO	Du Le Temple (10th century AD)
green	Emerald Green	Cu(CH_3_COO)_2_·3Cu(AsO_2_)_2_	Jiangxue Palace (15th century AD)
	Atacamite	Cu_2_(OH)_3_Cl	Longju Temple (15th century AD), etc.
	Phthalocyanine	CuC_32_N_8_Cl_16_	Prince Dai’s Mansion (19th century AD)
Blue	Lapis lazuli	(Na,Ca)_8_(AlSiO_4_)_6_(S,Cl)_2_	Du Le Temple (10th century AD)
	Azurite	2CuCO_3_·Cu(OH)_2_	Longju Temple (15th century AD)
	Indigo blue	C_16_H_8_N_2_Na_2_O_8_S_2_	Prince Dai’s Mansion (19th century AD)
White	Lead white	2PbCO_3_·Pb(OH)_2_	Puren Temple (18th century AD)
Black	Carbon black	C	Longju Temple (15th century AD), etc.

**Table 2:** Overview of techniques used for pigment analysis and elemental composition obtained by EDX analysis for respective pigment samples.

Sample	Color	Experimental Methods	Elements (Wt %)
DZ-1	Red	PLM, EDX, m-RS	Pb (57.8), Ba (20.6), Ca (6.6), Si (6.0), Ce (3.7)
DZ-2	Yellow	PLM, EDX, m-RS	Ca (32.5), Ba (18.9), Zn (16.9), Pb (16.7), Si (7.2), Cr (4.3)
DZ-3	Green	PLM, EDX, m-RS	As (35.9), Cu (33.1), Ca (11.5), Si (11.3), S (6.2)
DZ-4	Blue	PLM, EDX, m-RS	Ca (33.1), S (27.2), Si (25.2), Pb (7.6), K (3.0)
DZ-5	White	PLM, EDX, m-RS, FT-IR	Ca (33.4), S (22.1), Ba (19.9), Zn (8.5), Si (7.3), Pb (3.3)

**Table 3:** Component compositions of the yellow pigment sample (DZ-2).

Peak Number	Retention Time (min)	Area (%)	Compound
1	1.6057	0.26	Methylamine, N,N-dimethyl-
2	3.381	0.27	1H-Pyrrole, 1-methyl-
3	5.2443	0.04	glycine
4	5.2507	0.02	TMAH reagent
5	5.9213	0.44	1,3,5-Triazine, hexahydro-1,3,5-trimethyl-
6	6.1153	0.08	Alanine
7	7.873	0.01	valine
8	8.9157	0.06	L-Proline, 1-methyl-, methyl ester
9	10.237	0.05	Methyl 1-methylpyrrole-2-carboxylate
10	11.2533	0.04	Naphthalene
11	11.2567	0.06	1H-Indene, 1-methylene-
12	11.7583	0.06	Protein
13	11.8607	0.03	hydroxyproline
14	13.4727	2.99	Heptanedioic acid, dimethyl ester
15	14.8307	8.81	Octanedioic acid, dimethyl ester
16	16.127	38.56	Nonanedioic acid, dimethyl ester
17	17.279	3.09	Decanedioic acid, dimethyl ester
18	17.992	0.11	Protein—blood and glue
19	18.6543	1.44	Drying oil
20	20.2363	10.70	Hexadecanoic acid, methyl ester
21	22.164	8.95	Octadecanoic acid, methyl ester
22	22.409	0.27	Heat bodied oil
23	22.844	0.01	Benzene, isocyanato
24	24.1617	3.62	Drying oil
25	24.6707	0.29	Drying oil

## Data Availability

The datasets analyzed during the current study are available from the corresponding author upon reasonable request.
